# Optimization of quantitative reverse transcription PCR method for analysis of weakly expressed genes in crops based on rapeseed

**DOI:** 10.3389/fpls.2022.954976

**Published:** 2022-08-09

**Authors:** Michael Moebes, Heike Kuhlmann, Dmitri Demidov, Inna Lermontova

**Affiliations:** Leibniz Institute of Plant Genetics and Crop Plant Research (IPK), Gatersleben, Germany

**Keywords:** RT-qPCR, RNA isolation, RNA precipitation, cDNA synthesis, qPCR sensitivity, rapeseed, crops, low expression

## Abstract

Rapeseed (*Brassica napus*) is an allopolyploid hybrid (AACC genome) of turnip rape (*B. rapa*, genome: AA) and vegetable cabbage (*B. oleraceae*, genome: CC). Rapeseed oil is one of the main vegetable oils used worldwide for food and other technical purposes. Therefore, breeding companies worldwide are interested in developing rapeseed varieties with high yields and increased adaptation to harsh climatic conditions such as heat and prolonged drought. One approach to studying the mechanism of the epigenetically regulated stress response is to analyze the transcriptional changes it causes. In addition, comparing the expression of certain genes between stress- and non-stress-tolerant varieties will help guide breeding in the desired direction. Quantitative reverse transcription PCR (RT-qPCR) has been intensively used for gene expression analysis for several decades. However, the transfer of this method from model plants to crop species has several limitations due to the high accumulation of secondary metabolites, the higher water content in some tissues and therefore problems with their grinding and other factors. For allopolyploid rapeseed, the presence of two genomes, often with different levels of expression of homeologous genes, must also be considered. In this study, we describe the optimization of transcriptional RT-qPCR analysis of low-expression epigenetic genes in rapeseed, using *Kinetochore Null2* (*KNL2*), a regulator of kinetochore complex assembly, as an example. We demonstrated that a combination of various factors, such as tissue homogenization and RNA extraction with TRIzol, synthesis of cDNA with gene-specific primers, and RT-qPCR in white plates, significantly increased the sensitivity of RT-qPCR for the detection of *BnKNL2A* and *BnKNL2C* gene expression.

## Introduction

Rapeseed (*Brassica napus*) is a 30–150 cm tall herbaceous plant species of the cruciferous family (*Brassicaceae*). It is an allopolyploid hybrid (AACC genome) from a sexual cross between turnip rape (*B. rapa*, genome AA) and vegetable cabbage (*B. oleraceae*, genome CC). The number of chromosomes is 38, including 20 from *B. rapa* and 18 from *B. oleraceae*. According to Raymer (2002), this cross arose by chance through common cultivation, since no wild form could be found thus far. With a global annual production of 28.3 million tons in 2021/22, rapeseed oil is one of the most relevant vegetable oils in our society (Shahbandeh, 2022). Therefore, there is great interest not only in increasing the yield but also in adapting the plant as best as possible to different growing conditions. In this case, gene expression analyses play an important role, as they provide a snapshot of transcription under specific conditions in the analyzed plant tissue. Comparing the transcription of specific genes between the A and C genomes, as well as the transcription of specific genes in response to biotic or abiotic stress factors to which the plant is currently exposed, is of great interest. This would draw conclusions about the relevance of the genes and create targeted knockdown mutants that exhibit certain traits. A significant challenge in transcriptional analysis in rapeseed and other alloploid crops is the presence of several homologous sub-genomes, whose high total transcript pool generates the major difficulty of RT-qPCR analysis: low sensitivity for many weakly transcribed regulatory genes.

Analysis of gene expression usually involves many upstream processing steps, such as RNA extraction and purification, DNase digestion, and cDNA synthesis. However, often only general protocols are available, usually based on the model plant *Arabidopsis thaliana* (Oñate-Sánchez and Vicente-Carbajosa, 2008; Meng and Feldman, 2010). Moreover, regardless of the fact that *A. thaliana* and *B. napus* both belong to the same family *Brassicaceae*, they differ significantly in the content of plant secondary metabolites (Petersen et al., 2002; Fang et al., 2012), which has a considerable impact on sample preparation for RT-qPCR analysis. This study aimed to provide scientists with general technical approaches for increasing RT-qPCR sensitivity for the detection of weakly expressed genes in rapeseed crops.

This guide provides advice on cell disruption techniques, RNA isolation from frozen tissue, RNA precipitation and purification, cDNA synthesis strategies, and impact analyses of different upstream processing steps on the results of RT-qPCR measurements.

## Materials and methods

### Plant growth conditions

B*rassica* napus cv. Palma (wild type) plants were cultivated in a greenhouse with a 16-h light phase from 6 a.m. to 10 p.m. at a temperature of 20/18°C day/night and humidity level between 55 and 70%.

### RNA isolation

Root tissue material, rosette leaves, stem, stem leaves, buds, flowers, pollen, siliques, young embryos, and the upper part of the seedlings were collected in liquid nitrogen and distributed in 2 mL microcentrifuge tubes with two 1/8” stainless steel beads in equal amounts of approximately 150 mg. To compare tissue disruption efficiency, homogenization was performed using two different methods: (1) by grinding the frozen tissue in an MM400 CryoMill (Retsch, Haan, Germany) for 2 × 30 s at a frequency of 30 Hz; (2) by adding 100 μL TRIzol to the thawed samples before homogenization under the aforementioned conditions.

Total RNA was isolated from approximately 150 mg of tissue powder using two different methods. In the first method, isolation was performed using the RNeasy^®^ Plant Mini Kit (QIAGEN; Cat. No. 74904), according to the manufacturer’s instructions. In the second method, isolation was performed using TRIzol reagent (Thermo Fisher Scientific; Cat. No. 15596018). After homogenization of the samples in 100 μL TRIzol, an additional amount of 900 μL TRIzol was added for RNA extraction. In the regular protocol, samples were mixed with 1,000 μL TRIzol after homogenization. After thorough mixing and incubation of the samples at room temperature for 5 min, further extraction was performed according to the manufacturer’s instructions. The RNA pellet was dissolved in 40 μL RNase-free MilliQ-H_2_O. If the pellet did not completely redissolve or if co-precipitates were formed, the sample was frozen in liquid nitrogen for 2 min and then thawed directly at 28°C and 400 rpm in a shaker for 5 min. After centrifugation at 14,800 rpm and 4°C for 5 min, the supernatant was transferred to a new tube, and the pellet was discarded. For RNA isolation and subsequent RT-qPCR analysis, the materials listed in Supplementary Table 1 and equipment listed in Supplementary Table 2 were used.

### RNA concentration by precipitation

In case of low RNA yield, samples from the same tissues were pooled, precipitated, and resolubilized in smaller volumes. For precipitation, 0.1 times the sample volume, but not more than 20 μL, of 3 M sodium acetate and 1 μL of 2% glycogen were added to the sample. Samples were thoroughly mixed and centrifuged at 14,800 rpm and 4°C for 15 min, followed by addition of 2.5 times the sample volume of 96% ethanol. After centrifugation, the supernatant was discarded, and the pellet was washed once from all sides with 700 μL of 70% ethanol. The samples were then centrifuged at 14,800 rpm and 4°C for 5 min, and the supernatant was removed. Further, the pellet was pushed from the bottom to the inner wall of the tube with a pipette tip to ensure efficient evaporation of the residual solvent below the pellet. The pellet was dried in a shaker at 400 rpm at room temperature for 15 min, and the RNA was resolubilized with 40 μL of RNase-free MilliQ-H2O.

### Additional RNA purification

A portion of the TRIzol-extracts was additionally purified using NucleoSpin^®^ RNA Set for NucleoZOL (MACHEREY-NAGEL, Cat. No. 740406.50) according to the manufacturer’s instructions.

### Digestion by DNase and cDNA synthesis

To avoid genomic DNA contamination, RNA samples were treated with DNase using a Turbo DNA-free™ Kit (Thermo Fisher Scientific, Cat. No. AM1907) according to the manufacturer’s instructions. Concentration of the digested total RNA was adjusted to approximately 1 μg⋅μL^–1^. RNA concentration and purity were confirmed using the NanoDrop ND-1000 spectrophotometer, and RNA integrity was validated by 1% denaturing RNA gel electrophoresis in 1 × MOPS at 100 V for 30 min. Reverse transcription was performed using the first-strand cDNA synthesis kit (Thermo Fisher Scientific, Cat. No. K1622) according to the manufacturer’s instructions. For comparison, 100 pmol oligo (dT)_18_ or 20 pmol gene-specific antisense oligonucleotides were used for synthesis, together with 4 μg of RNA samples after DNase treatment. In order to assess the effectiveness of the DNase treatment, an RNA-qPCR was performed (Supplementary Figure 1).

### Quantitative reverse transcription PCR analysis

Standard quantitative reverse transcription measurements were performed using a full-skirted white 384-well plate (Biozym; Cat. No. 710892) and *TB Green Premix Ex Taq I* (TaKaRa; Cat. No. RR420A) using a QuantStudio 6 Flex system (Applied Biosystems, Thermo Fisher Scientific) according to the manufacturer’s instructions. For comparison, an additional measurement with a fully skirted, transparent 384-well plate (Biozym; Cat. No. 710872) was conducted. cDNA equivalent to 200 ng of total DNase-digested RNA and 5 pmol of each corresponding sense and antisense primer (Supplementary Table 3) were used in a 10 μL RT-qPCR reaction. The cycling conditions were as follows: 10 min of polymerase activation at 95°C, followed by 40 cycles at 95°C for 15 s, and 60°C for 1 min. The cycle threshold was manually set at 0.2. Each sample was represented by at least three technical replicates, which were analyzed during the same RT-qPCR run.

For preliminary interpretation of the amplification signal, the cycle threshold (Ct) raw data were used for comparison between the samples. Ct-values were transformed to 2^–ct^ without a calibration and normalization (Schmittgen and Livak, 2008). Validation of amplicon fragment size was performed by gel electrophoresis with 1.5% agarose in 1 × TAE at 100 V for 30 min and demonstrates the presence of only one distinct amplicon (Supplementary Figure 2). Melting curve analysis also shows the presence of only one distinct peak (Supplementary Figure 3), which indicates specific amplification.

### Determination of oligonucleotide-dependent amplification efficiency

To determine the oligonucleotide-dependent amplification efficiency, RNA from all available tissues was pooled, and gene-specific cDNA was synthesized under identical conditions as previously described. A 10-fold dilution series, ranging from 10^0^ to 10^–4^ was prepared using this cDNA mix. The dilution series was measured using the RT-qPCR method in white plates, as described above. Using the defined relative cDNA quantities and raw Ct data, a linear function was generated for each gene (Supplementary Figure 4). The slope of this function provides the basis for calculating the oligonucleotide-dependent amplification efficiency using the following equation: efficiency [%] = [10^(–1/slope)^ – 1] ⋅100 (Castonguay et al., 2015). The calculated efficiencies for *BnKNL2A* and *BnKNL2C*, as well as the efficiencies of the control oligos *BnACT7* and *BnUBC21* obtained from the original publications (Han et al., 2017), are listed in Supplementary Table 3.

### Statistical analysis

In all experiments, three to eight independent RNA samples were extracted for each variant. The replicates were then pooled into a single biological sample. Each sample was represented by at least three technical replicates, which were analyzed during the same RT-qPCR run. The cycle threshold (Ct) raw data transformed to 2^–ct^ were used for comparison between the samples (Supplementary Tables 4, 5). 2^–ct^ mean, standard error of 2^–ct^ mean, levels of significance (*p* < 0.05), and *p*-values were identified in frame of *t*-test analysis and descriptive statistics using GraphPad Prism version 8.0.1 for Windows^1^ (GraphPad Software, San Diego, California United States).

## Results

### Selection of reference and weakly expressed genes in rapeseed

To optimize the sensitivity of the RT-qPCR method, two classical housekeeping genes, *BnACT7* and *BnUBC21*, were selected as controls because they exhibit high specificity in RT-qPCR and similar levels of transcription in different tissues (Machado et al., 2015; Han et al., 2017). As a low-expression gene, we chose *BnKNL2*, an epigenetic regulator of kinetochore complex assembly and proper loading of centromeric histone H3 (CENH3) on the centromere. Comparative *in silico* analysis of *KNL2* gene transcription in *Arabidopsis* using *Arabidopsis* eFP Browser (Winter et al., 2007) showed that the highest expression of this gene was markedly lower than that of *AtACT7* and *AtUBC21* (58 vs. 2492 and 58 vs. 391, respectively). Below, we present several technical approaches that have been applied to increase the sensitivity of RT-qPCR for the analysis of the expression of genes with low levels of transcription.

### Homogenization of plant material with TRIzol increases RNA stability and sensitivity of quantitative reverse transcription PCR

**FIGURE 1:**
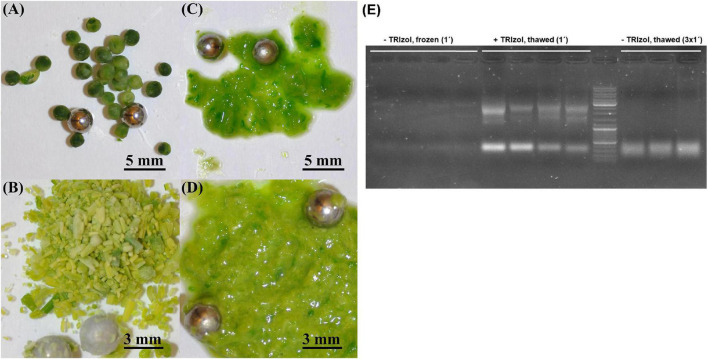
Comparison of different homogenization strategies in young seeds and buds. **(A)** Young seeds without TRIzol addition, **(B)** buds without TRIzol addition, **(C)** young seeds with addition of 100 μl TRIzol before homogenization, **(D)** buds with addition of 100 μl TRIzol before homogenization, **(E)** Impact of homogenization strategy on RNA integrity. Marker—Invitrogen gene ruler 1 kb + (*Cat. No. SM0311*); left—undegraded RNA (low yield) isolated without TRIzol addition before, 1 min of homogenization of frozen plant tissue; middle—undegraded RNA (high yield) isolated with addition of 100 μl TRIzol before, 1 min of homogenization of thawed plant tissue; right—degraded RNA (high yield) isolated without TRIzol addition before, 3 × 1 min of homogenization in order to reach comparable tissue disruption efficiency in comparison with + TRIzol samples.

**FIGURE 2:**
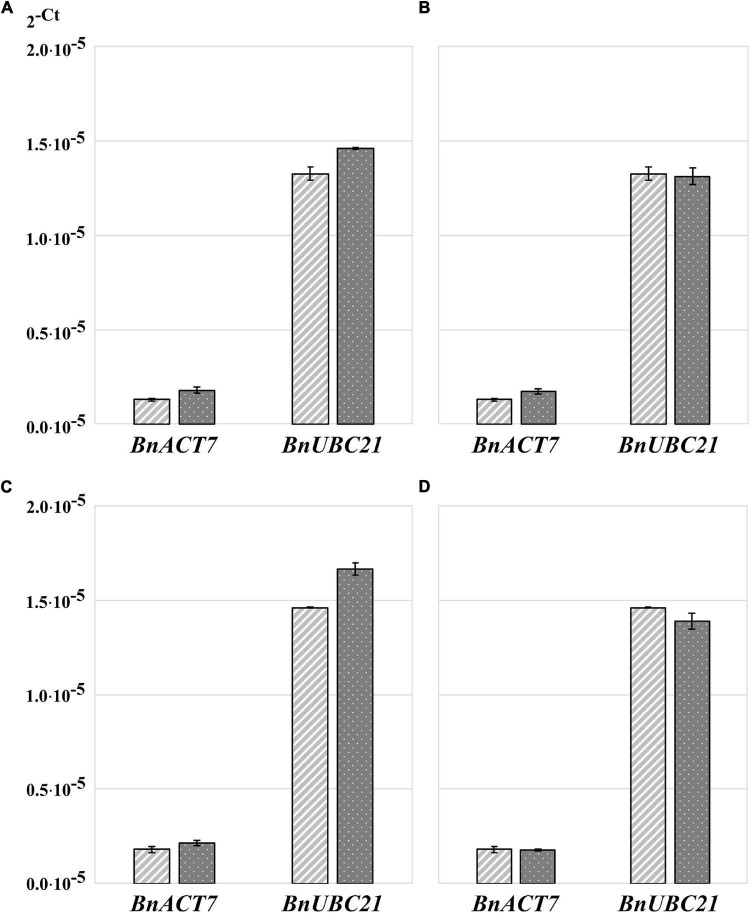
Transformed cycle-threshold (2^– ct^) values of *ACT7* and *UBC21* transcripts in flower buds of *B. napus* (*v. Palma*). **(A)** Influence of sample homogenization without and with TRIzol™ addition on qPCR performance, light gray—samples without addition of TRIzol™ before homogenization, dark gray—samples with TRIzol addition. **(B)** Influence of RNA-isolation method on qPCR performance, light gray—samples isolated with TRIzol™- Reagent, dark gray—samples isolated with RNeasy^®^ plant mini kit. **(C)** Influence of RNA precipitation on qPCR performance, light gray—unprecipitated RNA, dark gray—precipitated RNA. **(D)** Influence of additional RNA purification on qPCR performance, light gray –TRIzol™ RNA without additional purification, dark gray—additionally purified RNA using NucleoSpin^®^ RNA Set for NucleoZOL after TRIzol™ isolation. Error bars: standard deviation of sample (N-1) *N* = 4.

Because RNA yield and stability are key factors in RNA isolation methods, we tested whether homogenization of plant material with TRIzol would increase the yield of RNA and/or reduce its degradation. The high guanidine thiocyanate and acid phenol content of TRIzol leads to the denaturation of membrane proteins. Consequently, TRIzol penetrates the cells before homogenization and ensures efficient RNase inhibition, whereas without the addition of TRIzol, RNases can become active in the cells as soon as the samples begin to thaw (Meng and Feldman, 2010). Thus, in the control samples, the tissue was homogenized immediately after its removal from liquid nitrogen (Figures 1A,B). In the case of the test variants, 100 μL of TRIzol was added to the samples immediately after removal from liquid nitrogen, and the samples were then thawed on ice for 2–3 min before homogenization (Figures 1C,D). We found that homogenization of plant tissue without the addition of TRIzol resulted in an RNA yield of 0.176 μg⋅μL^–1^, with an A260/A280 ratio of 1.825 and A260/A230 ratio of 0.45, whereas homogenization of plant tissue with 100 μL of TRIzol resulted in an RNA yield of 1.67 μg⋅μL**^–^**^1^, A260/A280 ratio of 1.95, and A260/A230 ratio of 0.775. Thus, homogenization of samples in the presence of TRIzol resulted in a strong increased RNA concentration because addition of TRIzol strongly increased the quality of homogenization (Figures 1C,D) and RNA stability (Figure 1E). These two aspects are very important for rapeseed, which, in contrast to *Arabidopsis*, has larger plant organs, with some having high water content (young seeds, flower buds, embryos). Additionally, many tissues are coarser than those of *Arabidopsis*, such as stems, old leaves, and roots. In most cases, such frozen samples can hardly be homogenized (Figures 1A,B), but if thawed without TRIzol, they run the risk of RNA degradation (Figure 1E). Because of this feature, all further RNA extractions were performed by homogenizing the samples in the presence of TRIzol (Figures 1C,D). To test the influence on RT-qPCR sensitivity, the amplification of specific cDNA using RNA samples homogenized with and without TRIzol was compared using two selected housekeeping genes, *BnACT7* and *BnUBC21*. A comparison of the amplification data (Figure 2A) showed that for both *BnACT7* and *BnUBC21*, the 2^–ct^ values were significantly higher (Supplementary Table 4) for the samples homogenized with TRIzol. Hence, the TRIzol isolation method not only avoids RNA degradation but also increases RT-qPCR sensitivity.

### Quantitative reverse transcription PCR results were not influenced by RNA isolation method

Total RNA isolation was performed using two methods: the first using TRIzol and the second using the RNeasy kit. A comparison of the amplification data (Figure 2B) showed that the 2**^–ct^** value of *BnACT7* was not strong but significantly higher (Supplementary Table 4) when RNA was isolated with the RNeasy kit than that of TRIzol samples with the same amount of RNA. For *BnUBC21*, no influence of isolation method was detected (Figure 2B and Supplementary Table 4). Thus, the use of RNeasy spin columns has no obvious advantages over the TRIzol method. Moreover, the RNA extraction procedure using TRIzol is faster and less expensive. However, we have to point out that the use of TRIzol does not allow isolation of undegraded RNA from all types of tissue; the typical problematic tissues for isolation with TRIzol are stems, seedlings, and siliques.

### RNA concentration and purity can be increased by RNA precipitation

In some tissues, such as stems, leaves, and siliques, the RNA yield after extraction is lower than optimal for cDNA synthesis to detect transcripts from genes with low expression levels. In such cases, RNA extracts were first pooled, precipitated, and then re-dissolved in a smaller volume. This precipitation process resulted in a loss of 1.48% (65.41 ng⋅μL**^–^**^1^) of total RNA compared to the primary TRIzol RNA extract used for analysis. The A260/A280 ratio increased from 1.30 to 1.39 (6.47%), and the A260/A230 ratio increased from 1.22 to 1.45 (15.86%) due to precipitation. Comparison of the amplification data showed significantly higher 2**^–ct^** values for the precipitated compared to the untreated samples (Figure 2C and Supplementary Table 4) when using the same RNA amount for cDNA synthesis, which can be correlated with lower contamination of the sample with proteins or organic solvents.

Since the RNA samples after TRIzol extraction were not of high purity, we assumed that an additional purification of RNA on silica membrane columns could increase the sensitivity of RT-qPCR. After TRIzol extraction, one part of the RNA sample was purified using the NucleoSpin RNA Set for NucleoZOL (MACHEREY-NAGEL). The yield of the first elution of purified RNA was 54.4% (2.39 μg⋅μL**^–^**^1^) of total TRIzol RNA extract; further elution steps were not analyzed. The A260/A280 ratio of the first elution was increased from 1.34 to 2.14 (62.6%), and the A260/A230 ratio increased from 1.21 to 2.23 (54.3%). Interestingly, amplification data for *BnUBC21* shows significantly lower 2**^–ct^** values, when samples were purified after TRIzol extraction, while for *BnACT7*, no significant differences were detected (Figure 2D and Supplementary Table 4). Consequently, although additional purification of the RNA samples clearly increased the A260/A280 and A260/A230 ratios, it did not increase the sensitivity of the method.

### Quantitative reverse transcription PCR sensitivity can be increased by using gene-specific nucleotides for cDNA synthesis

**FIGURE 3:**
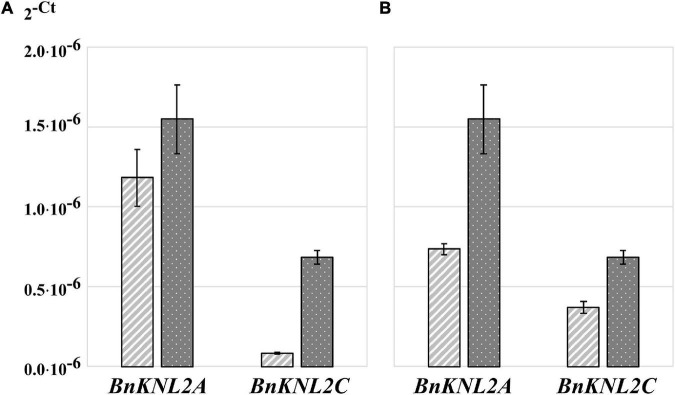
Transformed cycle-threshold (2^– ct^) values of *BnKNL2A* and *BnKNL2C* transcripts in a cDNA-mix of all analyzed tissues of *B. napus* (*v. Palma*). **(A)** Influence of cDNA synthesis strategy on qPCR sensitivity, light gray—cDNA, synthetized with unspecific oligo (dT)_18_, dark gray–specific cDNA, synthetized with antisense oligonucleotides of the corresponding gene. **(B)** Influence of different qPCR plates on signal intensity, light gray—transparent plate, dark gray—white plate of the same manufacturer. Error bars: standard deviation of sample (N-1) *N* = 3.

To analyze the effects of different approaches of sample homogenization, RNA isolation, concentration and purification on RNA quality, quantity, and concentration, RT-qPCR was performed with control primers. However, even with the highest RNA quality and quantity, it is not always easy to detect gene transcripts with low expression, particularly in polyploid crop species. One option for increasing the sensitivity of RT-qPCR is the use of gene-specific oligonucleotides for cDNA synthesis. This method leads to a strongly reduced amount of non-specific sample template, as only the cDNA of the gene of interest is present as a substrate for RT-qPCR analysis. Therefore, we compared the sensitivity of RT-qPCR for cDNA samples synthesized either with non-specific oligo (dT)_18_ or with specific *BnKNL2C* and *BnKNL2C* oligonucleotides. The results of the RT-qPCR analysis showed that in the case of the *BnKNLC* gene, a significantly higher amplification sensitivity was observed for specific cDNA than that for the control oligo (dT)_18_ cDNA (Figure 3A and Supplementary Table 4). However, in the case of B*nKNL2A*, the difference between specific and non-specific cDNA was not very strong (Figure 3A and Supplementary Table 4). Nevertheless, there was a clear tendency toward better amplification of the specific cDNA.

### Sensitivity of quantitative reverse transcription PCR analysis of low-expression genes is strongly influenced by plate material

Another method to enhance RT-qPCR sensitivity is to select suitable RT-qPCR plates. A common problem during RT-qPCR analysis is the high cycle threshold value for genes with low expression, which may result in unreliable data. In such cases, a signal with a low intensity should be detected as sensitively as possible. An important and often underestimated point for the accurate and sensitive detection of fluorescence in RT-qPCR is the choice of suitable plates. Transparent plates are used for RT-qPCR analysis, but recently, many companies have started manufacturing RT-qPCR-specific white plates, claiming that they reflect more signals back to the detector than transparent plates, thereby increasing the signal-to-noise ratio. Comparison of white and transparent plates manufactured by the same company showed that the signal intensity for amplification of *BnKNL2* genes from the same sample was significantly higher in all cases when using white plates than that with transparent plates (Figure 3B and Supplementary Table 4).

### A combination of optimal methodological approaches significantly increases the amplification efficiency of the low-expressed *Kinetochore null2* genes

Next, we tested the additive effect of the above-mentioned specific cDNA synthesis and the use of white plates on the amplification sensitivity of low-expression *BnKNL2* genes.

Homogenization and RNA isolation for all samples were performed based on the optimizations described above. First, tissues were homogenized with the addition of 100 μL TRIzol to increase the RNA yield and stability. Second, with the exception of the silique, all tissue samples were isolated using the TRIzol reagent. RNA from silique samples was isolated using RNeasy spin columns, as the use of TRIzol for this tissue type results in RNA degradation. Third, in the case of samples isolated from stems, rosette leaves, and siliques, a high yield of RNA (4 μg in 11 μL), could be achieved only after additional concentration of RNA by precipitation.

**FIGURE 4:**
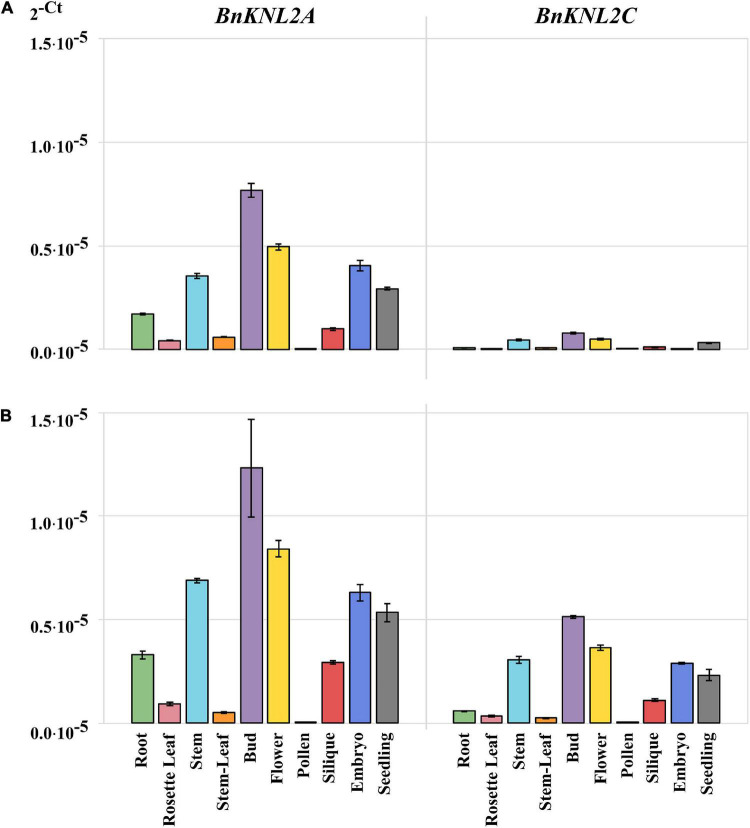
Comparison of transformed cycle-threshold (2^– ct^) values of *BnKNL2A* and *BnKNL2C* transcripts in all analyzed tissues of *B. napus* (*v. Palma*). **(A)** cDNA, synthetized with unspecific oligo (dT)_18_ and measured in a transparent qPCR plate. **(B)** Specific cDNA, synthetized with antisense oligonucleotides of the corresponding gene and measured in a white qPCR plate. Error bars: standard deviation of sample (N-1) *N* = 3.

We then compared the RT-qPCR results obtained on transparent plates using cDNA samples synthesized with oligo (dT)_18_ (Figure 4A) with those obtained on white plates with specific cDNA (Figure 4B). For both *BnKNL2A* and *BnKNL2C*, significantly higher signal intensity was detected in all tissues, except for *BnKNL2A* in stem leaves, when gene-specific cDNA was used in combination with a white plate in comparison to unspecific cDNA in combination with a transparent plate (Supplementary Table 5). In summary, the combination of different optimization steps of the RT-qPCR method can significantly increase the sensitivity and reliability of the expression data, especially for the analysis of low-transcription genes in crops.

## Discussion

RT-qPCR-based transcriptional analysis is a powerful tool to provide important information about gene activity in different organs/tissues at a specific stage of plant development. It is well known that the transcription levels of different genes in an organism vary significantly (Klepikova et al., 2016). Studies on the activity of genes with high transcription levels are well established not only for model plants but also for many crops (Salihah et al., 2016; Yan et al., 2019; Zhang et al., 2019; Bai et al., 2022). However, transcriptional analysis of weakly active genes in crops is not trivial. An important part of such an analysis is the isolation of sufficient amounts of high-quality RNA from different tissues. We encountered various challenges in isolating RNA from different rapeseed tissues. Plant RNA samples often contain contaminants as co-precipitates (Kansal et al., 2008; Wang et al., 2012). In particular, RNA from seeds, pollen, or siliques is colored and contaminated with phenols and/or polysaccharides (Suzuki et al., 2004; Li and Trick, 2005; Allorent et al., 2010). Another problem is the limited amount of material and difficulty in collecting it, as in the case of young seeds (Footitt et al., 2018) or embryos. These technical problems make RT-qPCR analysis of weakly transcribed genes even more challenging. Finally, obtaining reliable transcription data for weakly expressed genes is limited by the sensitivity of the RT-qPCR analysis. For this reason, we present some simple modifications of the RT-qPCR method that will help increase the sensitivity of the assay and can be used for crops, even for difficult tissue samples. The critical point for obtaining RNA with high yield and good quality by extraction from plant material is grinding. The samples were homogenized as thoroughly and as strongly as possible. However, during homogenization, samples are heated, which causes RNA degradation. Additionally, homogenization releases endogenous nucleases, which further degrade RNA. The best compromise is to homogenize the cooled sample in the presence of nuclease-inactivating agents such as chaotropic salts or phenol (Chomczynski and Sacchi, 1987).

Because TRIzol contains phenol and chaotropic salts (Chomczynski and Sacchi, 1987), the chance of degradation during cell disruption is distinctly lower compared to untreated samples. This is especially important when the sample material is difficult to obtain and/or frozen samples cannot be homogenized. Furthermore, RT-qPCR analysis showed a significantly higher signal level for both control genes (*BnACT7* and *BnUBC21*) from samples homogenized with TRIzol compared to samples without prior TRIzol addition. Thus, the addition of TRIzol before homogenization of the plant material is recommended as it increases the RNA stability and sensitivity of RT-qPCR.

For a long time, we successfully used the TRIzol method for RNA isolation from various tissues and organs of different plants. Compared to other commercially available methods of plant RNA isolation, TRIzol is inexpensive in terms of cost per sample. In addition, several TRIzol-similar reagents are available from different companies: TRIzol (Invitrogen), QIAzol (Qiagen), TRI Reagent and RNAzol (Sigma-Aldrich), TRIpure Reagent (Aidlab), and TriSure (Bioline). However, the use of TRIzol to isolate RNA from some tissue types, such as stems, seedlings and siliques, may result in its degradation.

The reason can be specific for each tissue type. For instance: young seedlings usually contain large amounts of RNases and nucleases required for nucleotide remobilization; stems are cellulose-rich and contain dead xylem tissue; problems of RNA isolation from siliques most likely are related to the high content of polysaccharides and phenols (Oñate-Sánchez and Vicente-Carbajosa, 2008).

Therefore, RNA was isolated from these three tissues using the QIAGEN RNeasy^®^ plant mini kit, although this method is more expensive than TRIzol-based methods. The differences in sample purity in favor of column isolation, as we have observed, did not negatively influence the effectiveness of RT-qPCR because a slight increase in RT-qPCR sensitivity for *BnACT7* was not confirmed for the *BnUBC21* gene. A similar effect for *GAPDH* and lamin A has already been demonstrated in RNA extracts from mouse livers (Mandrekar, 2008). Therefore, in the case of column-based RNA isolation, it needs to be determined whether the loss of RNA due to limited RNA column binding is critical. From our point of view, a more reasonable method is the combination of RNA extraction with TRIzol and precipitation of RNA for samples low concentration or purity, as described in previous sections (refer to Methods and Results). To detect transcripts of weakly expressed genes by RT-qPCR, cDNA synthesis must be performed with a sufficient amount of highly concentrated RNA. However, depending on the tissue, the concentration of RNA in individual samples may vary greatly and is below the amount required for synthesis. In such cases, complete re-extraction of samples is not always possible because of the limited amount of plant material. Therefore, we propose combining several replicates, followed by precipitation and re-dissolution of RNA in smaller volumes. In addition to increasing the concentration, this approach can efficiently remove contaminants and generate RNA with a quality comparable to that obtained with column-based RNA purification kits. Thus, it helps minimize the costs and time required for RNA isolation. In addition, the mechanical impact on RNA during column-based purification is higher than that during RNA precipitation. For both *BnACT7* and *BnUBC21*, the signal intensity during RT-qPCR analysis was significantly higher when RNA was precipitated than when it was not. This result may be attributed to the fact that after RNA precipitation, the amount of contaminants (Vicient and Delseny, 1999; Demeke and Jenkins, 2010) is reduced, and thus, the inhibition of reverse transcriptase or DNA polymerases is no longer strong. This resulted in a better amplification efficiency and an increase in RT-qPCR sensitivity.

However, we found that purification of TRIzol-isolated RNA through a column did not increase the sensitivity of RT-qPCR, as expected, and there was no significant difference in signal intensity between purified and uncleaned samples in the case of *BnACT7*, whereas in the case of *BnUBC21*, the opposite tendency was observed. One reason for this effect could be that additional purification *via* the column not only removes proteins but also adds new contaminants such as chaotropic polymerase inhibitory salts. Thus, the simultaneous concentration and purification of RNA using RNA precipitation is a preferable approach to RNA purification using columns.

Because RT-qPCR sensitivity strongly depends on the cDNA synthesis strategy, we compared the sensitivity of RT-qPCR for cDNA samples synthesized with either gene-specific or oligo (dT)_18_ primers. We showed that in the case of low-expression *BnKNL2C*, the specific cDNA demonstrated significantly higher signal intensities than oligo (dT)_18_ cDNA (Figure 3A and Supplementary Table 4). A similar, but less pronounced, tendency was observed for *BnKNL2A* (Figure 3A and Supplementary Table 4). This result is in line with our expectations as only cDNA was synthesized with gene-specific primers and used as a template for RT-qPCR. In the case of cDNA synthesized with oligo (dT)_18_, the template sample consisted of large amounts of total non-specific cDNA, which made amplification of the specific transcript more difficult and reduced RT-qPCR sensitivity.

An often-underestimated factor influencing RT-qPCR measurements is the selection of suitable plates. Particularly in the case of weak signal intensities, as in the case of low-expressed genes, it is important to detect the signal without loss and as precisely as possible. We found that the signal intensity was significantly higher for the amplification of *BnKNL2A* and *BnKNL2C* transcripts when white plates were used for RT-qPCR than that when transparent plates were used. This observation also corresponds to the information provided by Thermo Fisher.^2^ This result is attributed to the lower scattering of SYBR-Green and ROX signals in white plates. The fluorescence reaches the detector bundled because the signal is reflected inside the wells. In the case of transparent plates, a considerable part of the signal is lost owing to the superior transmission properties of the material.

Using gene-specific oligonucleotides for cDNA synthesis and white plates strongly increases the sensitivity of RT-qPCR and allows the detection of transcripts of low-expression genes, even in tissues with transcript levels below the detection limit or with high Ct values that cannot be quantified properly. Even for samples within an acceptable range of Ct < 30, it is advantageous to lower Ct values to reduce variation between replicates and clarify the difference with a non-template control to increase the reliability of the results.

To demonstrate the influence of the two factors, the cDNA synthesis strategy and the choice of RT-qPCR plates, tissue-specific analysis of the two low-transcription genes *BnKNL2A* and *BnKNL2C* was performed. The first measurement was performed under standard conditions using transparent plates and non-specific cDNA synthesized with oligo (dT)_18_ and the second, according to our recommendations, with white plates and gene-specific cDNA. In principle, both *BnKNL2A* and *BnKNL2C* showed a significant increase in the RT-qPCR signal when a combination of specific cDNA and white plates was used.

The detection of the transcriptional levels of both genes in different tissues improved significantly when our recommendations were used, but the strongest effect was found for the *BnKNL2C* gene. In particular, the transcription level of *BnKNL2C* is so weak that it is nearly impossible to determine its expression level in different tissues using the standard RT-qPCR protocol. By using our recommended changes to the RT-qPCR protocol, it was possible to increase detection sensitivity and, in general, accurately determine the real expression level of *BnKNL2C* in most tissues.

In summary, the following strategy has proven to be optimal:

1. Sample homogenization is recommended with the addition of 100 μL TRIzol per 150–200 mg tissue, as it provides a homogeneous sample, stabilizes RNA, and increases RNA yield.
2. To achieve high RNA yield, RNA isolation using TRIzol is recommended without additional purification of the RNA sample on spin columns. However, in some tissue types, such as stems, seedlings and siliques, TRIzol RNA may degrade; in this case, column-based isolation can be performed. RNA precipitation is recommended to increase the RNA purity and concentration.
3. To improve the amplification of weakly expressed genes, specific cDNA synthesis instead of oligo (dT)_18_ is recommended.
4. The use of white instead of transparent plates significantly increased the signal intensity, which improved the detection of weakly expressed genes.

The most successful option in such cases is to combine several approaches, for example, the addition of TRIzol by sample homogenization, precipitation of RNA when its concentration is low, synthesis of cDNA with a specific primer, and use of white plates for RT-qPCR.

## Data availability statement

The original contributions presented in the study are included in the article/Supplementary material, further inquiries can be directed to the corresponding author/s.

## Author contributions

MM, DD, and IL conceived the study, designed the experiments, and wrote the manuscript. MM and HK performed the experiments. All authors have read and approved the final manuscript.

## Footnotes

1
www.graphpad.com

2
https://www.thermofisher.com/de/de/home/life-science/cloning/cloning-learning-center/invitrogen-school-of-molecular-biology/pcr-education/pcr-qpcr-plastics/pcr-qpcr-plastics-considerations.html
