# Supplementary material for: Optimization of quantitative reverse transcription PCR method for analysis of weakly expressed genes in crops based on rapeseed
Source: Front Plant Sci. 2022 Aug 9;13:954976. doi: 10.3389/fpls.2022.954976 (PMC9396215; doi:10.3389/fpls.2022.954976)
Supplement: Supplementary file 1 [file Data_Sheet_1.PDF]

Supplementary Material

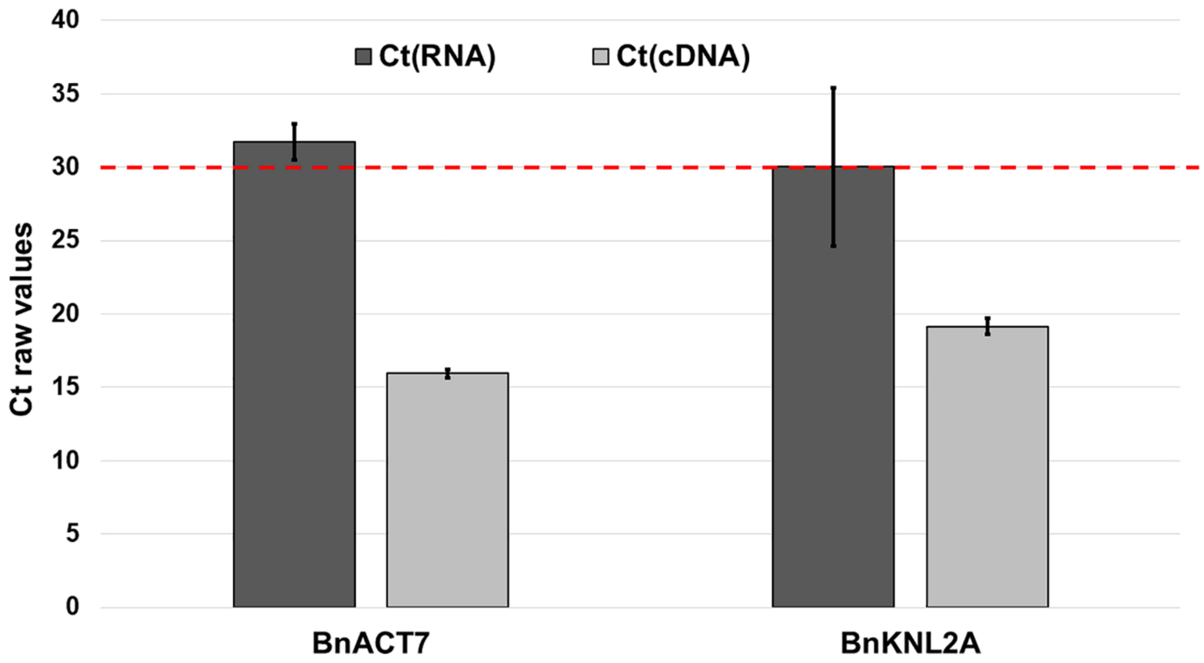

Supplementary Figure S1. Determination of DNase digestion efficiency using RT-qPCR. Dark grey – RNA as template, amplified with *BnACT7* and *BnKNL2A* oligonucleotides. Light grey – specific cDNA as template, amplified with *BnACT7* and *BnKNL2A* oligonucleotides. Red line: Limit of quantification after 30 cycles.

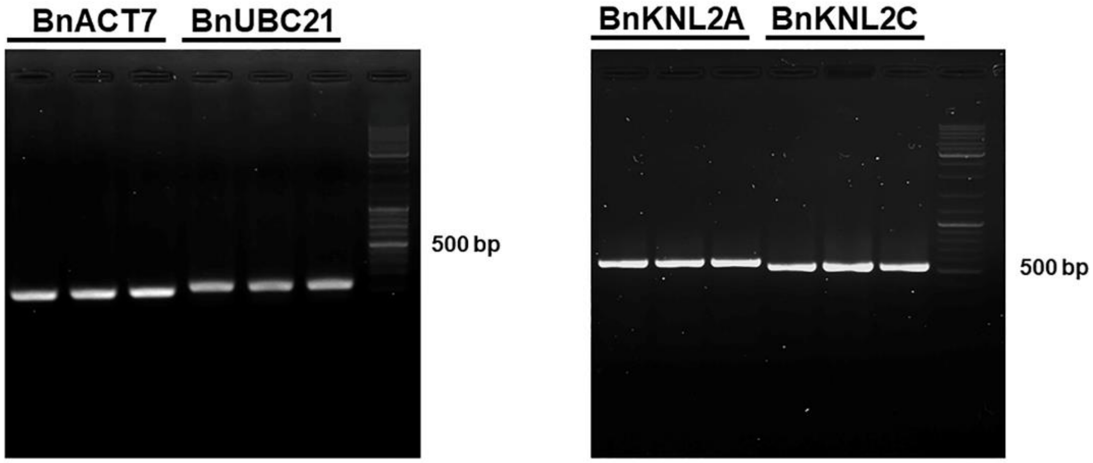

Supplementary Figure S2. Gelelectrophoresis of RT-qPCR products on 1.5 % gel - *BnACT7* (148 bp), *BnUBC21* (176 bp), *BnKNL2A* (579 bp) and *BnKNL2C* (566 bp).

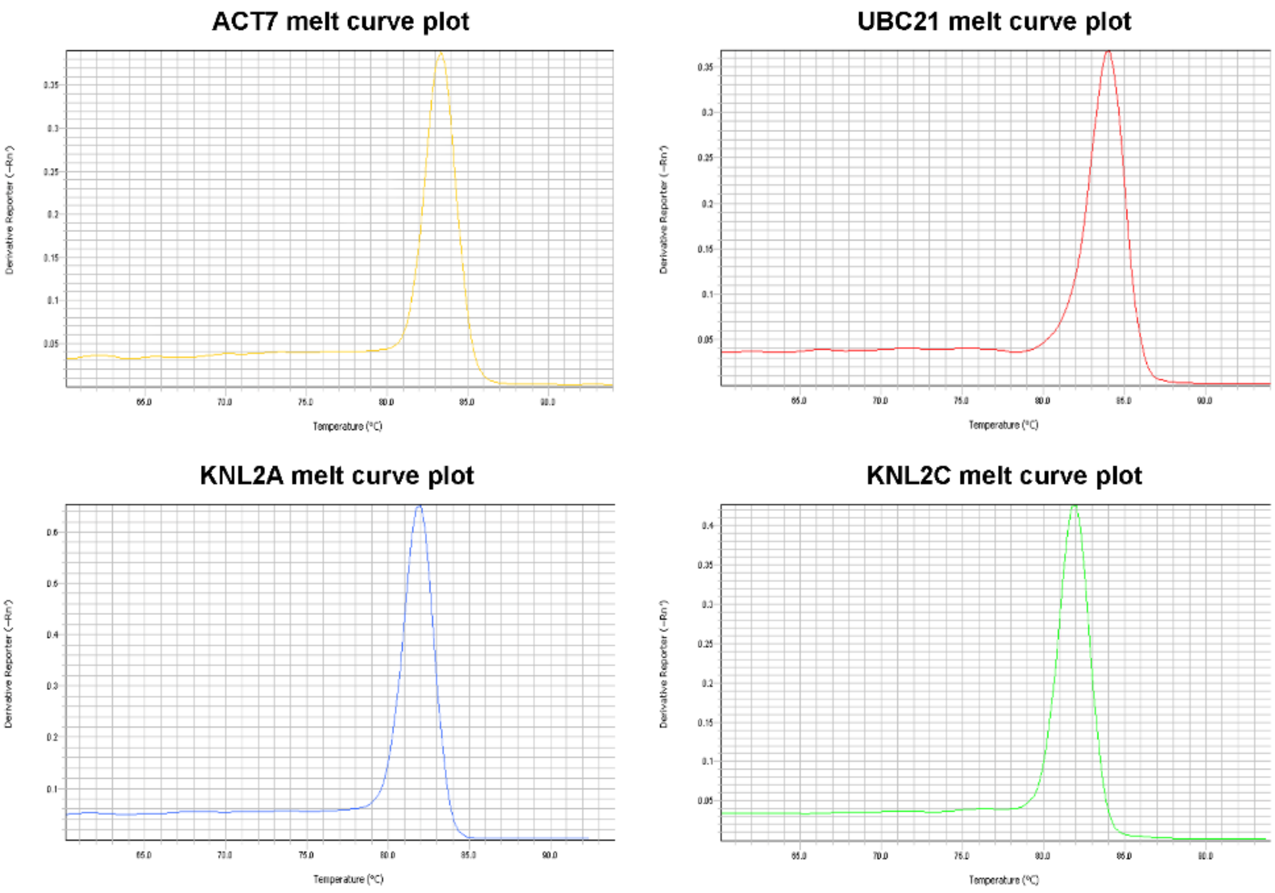

Supplementary Figure S3. Melt curve plot of: *BnACT7* (top left, yellow), *BnUBC21* (top right, red), *BnKNL2A* (bottom left, blue) and *BnKNL2C* (bottom right, green) using specific root cDNA.

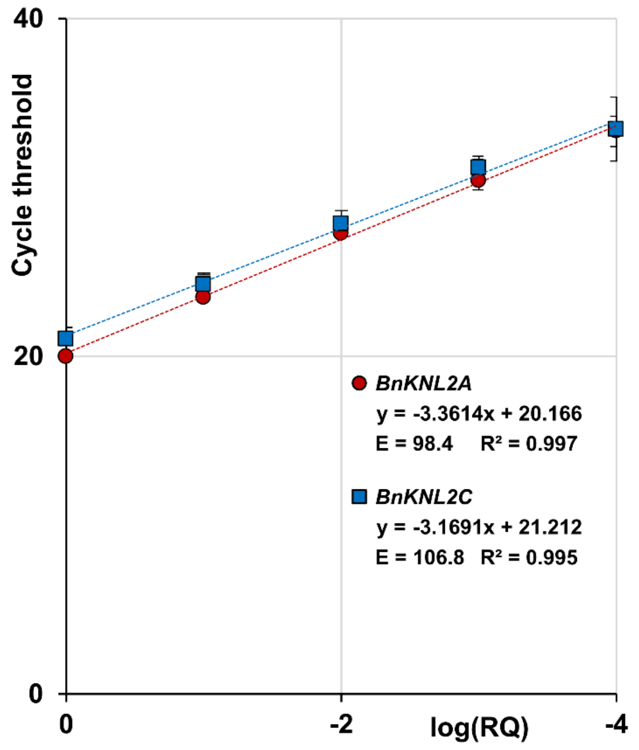

Supplementary Figure S4. Determination of the oligonucleotide-specific amplification efficiency. Linear function: the dependence of the cycle threshold on the logarithm of the relative quantity. Red – *BnKNL2A*, Calculated Efficiency [%]: 98.4. Blue – *BnKNL2C*, Calculated Efficiency [%]: 106.8. Error bars represent standard deviation of sample.

Supplementary Table S1. Materials

Reagent* or Kit†	Cat. No.	Manufacturer
Biozym LE Agarose*	840001	Biozym Scientific GmbH (Hessisch Oldendorf, DE)
Ethanol*	T171.1	Carl Roth GmbH + Co. KG (Karlsruhe, DE)
Glycogen (mol. biol. grade) *	R0561	Thermo Fisher Corp. (Waltham, USA)
Intas HDGreen™ Plus DNA-Dye*	N/A	Intas Science Imaging Instruments GmbH (Göttingen, DE)
Isopropanol*	9866.5	Carl Roth GmbH + Co. KG (Karlsruhe, DE)
NucleoSpin® RNA Set for NucleoZOL†	740406.50	Macherey-Nagel GmbH & Co. KG (Düren, DE)
RevertAid First Strand cDNA Synthesis Kit†	K1621	Thermo Fisher Corp. (Waltham, USA)
RNeasy® Plant Mini Kit†	74904	Qiagen N.V. (Hilden, DE)
Sodium acetate trihydrate*	3856.1	Carl Roth GmbH + Co. KG (Karlsruhe, DE)
TB Green™ Premix Ex Taq™ I (Tli RNase H Plus) †	RR420A	Takara Bio Inc. (Kusatsu, JP)
TRIzol™ Reagent*	15596018	Thermo Fisher Corp. (Waltham, USA)
TURBO DNA-free™ Kit†	AM1907	Thermo Fisher Corp. (Waltham, USA)

Supplementary Table S2. Equipment

Instrument	Model	Manufacturer
Centrifuge	Megafuge 8	Thermo Fisher Corp. (Waltham, USA)
Gel Documentation	Gel Stick Touch	Intas Science Imaging Instruments GmbH (Göttingen, DE)
Microcentrifuge	Fresco™ 21	Thermo Fisher Corp. (Waltham, USA)
Spectrophotometer	Nanodrop ND-1000	Thermo Fisher Corp. (Waltham, USA)
Pipette Set	Pipetman Classic	Gilson Inc. (Middleton, USA)
Real-Time PCR System	QuantStudio 6 Flex system	Thermo Fisher Corp. (Waltham, USA)
ThermoShaker	Biometra TS1	Analytik Jena GmbH (Jena, DE)
Vortex mixer	Vortex-Genie™ 2	Thermo Fisher Corp. (Waltham, USA)

Supplementary Table S3. Oligonucleotide sequences and amplification efficiencies

Oligonucleotide	Sequence [5‘-3‘]	length [bp]	GC [%]	Tm [°C]	E	R^2
BnACT7_fw	CCTCTCAACCCGAAAGCGAA	20	55.0	59.3	0.967[A]	0.997[A]
BnACT7_rev	CATCACCAGAGTCGAGCACA	20	55.0	59.3	0.967[A]	0.997[A]
BnUBC21_fw	TATCCTCTGCAGCCTCCTCA	20	60.0	61.4	1.002[A]	0.995[A]
BnUBC21_rev	CTGTCTGCCTCAGGATGAGC	20	60.0	61.4	1.002[A]	0.995[A]
BnKNL2A_fw	AACGACAAGAAACGGAATCTAGAGGA	26	42.3	61.6	0.984[B]	0.997[B]
BnKNL2A_rev	AGTCGTCGTCATAGGCTCTACTACTA	26	46.2	63.2	0.984[B]	0.997[B]
BnKNL2C_fw	GCACTGTCACTGCTAAGAAGAAGAAG	26	46.2	63.2	1.098[B]	0.995[B]
BnKNL2C_rev	CTTGGAGGACCTTCAATGAGTAGTCA	26	46.2	63.2	1.098[B]	0.995[B]

Purification: HPSF. Manufacturer: Eurofins Genomics Germany GmbH, 85560 Ebersberg, Germany. [A] (Han et al., 2017), [B] Efficiencies calculated by the slope of the corresponding linear function.

Supplementary Table S4. Statistical analysis of transformed cycle-threshold (2^{-Ct}) values for each method development step

Table 4: mean 2^{-Ct} values, standard error of mean, p-values and significance of method development steps

Assay	Target	Treatment	2^{-Ct} mean	Standard error of 2^{-Ct} mean	p-value	significant ($p < 0.05$)?
homogenization	*ACT7*	- TRIzol<br>+ TRIzol	$1.3 \cdot 10^{-06}$<br>$1.8 \cdot 10^{-06}$	$4.4 \cdot 10^{-08}$<br>$8.4 \cdot 10^{-08}$	0.0019	Yes
	UBC21	- TRIzol<br>+ TRIzol	$1.3 \cdot 10^{-05}$<br>$1.5 \cdot 10^{-05}$	$1.7 \cdot 10^{-07}$<br>$1.5 \cdot 10^{-08}$	0.0002	Yes
isolation	*ACT7*	TRIzol<br>Column	$1.3 \cdot 10^{-06}$<br>$1.7 \cdot 10^{-06}$	$4.4 \cdot 10^{-08}$<br>$6.4 \cdot 10^{-08}$	0.0011	Yes
	UBC21	TRIzol<br>Column	$1.3 \cdot 10^{-05}$<br>$1.3 \cdot 10^{-05}$	$1.7 \cdot 10^{-07}$<br>$2.2 \cdot 10^{-07}$	0.7304	No
precipitation	*ACT7*	n. precipitated<br>precipitated	$1.8 \cdot 10^{-06}$<br>$2.1 \cdot 10^{-06}$	$8.4 \cdot 10^{-08}$<br>$7.4 \cdot 10^{-08}$	0.0181	Yes
	UBC21	n. precipitated<br>precipitated	$1.5 \cdot 10^{-05}$<br>$1.7 \cdot 10^{-05}$	$1.5 \cdot 10^{-08}$<br>$1.7 \cdot 10^{-07}$	<0.0001	Yes
purification	*ACT7*	n. purified<br>purified	$1.8 \cdot 10^{-06}$<br>$1.8 \cdot 10^{-06}$	$8.4 \cdot 10^{-08}$<br>$2.4 \cdot 10^{-08}$	0.9671	No
	UBC21	n. purified<br>purified	$1.5 \cdot 10^{-05}$<br>$1.4 \cdot 10^{-05}$	$1.5 \cdot 10^{-08}$<br>$2.1 \cdot 10^{-07}$	0.0144	Yes
cDNA synthesis	*KNL2A*	oligo(dT)18<br>specific	$1.2 \cdot 10^{-06}$<br>$1.5 \cdot 10^{-06}$	$1.0 \cdot 10^{-07}$<br>$1.2 \cdot 10^{-07}$	0.0927	No
	KNL2C	oligo(dT)18<br>specific	$8.5 \cdot 10^{-08}$<br>$6.8 \cdot 10^{-07}$	$3.8 \cdot 10^{-09}$<br>$2.4 \cdot 10^{-08}$	<0.0001	Yes
qPCR plates	*KNL2A*	transparent<br>white	$7.6 \cdot 10^{-07}$<br>$1.5 \cdot 10^{-06}$	$2.1 \cdot 10^{-08}$<br>$1.2 \cdot 10^{-07}$	0.0027	Yes
	KNL2C	transparent<br>white	$4.0 \cdot 10^{-07}$<br>$6.8 \cdot 10^{-07}$	$2.0 \cdot 10^{-08}$<br>$2.4 \cdot 10^{-08}$	0.0007	Yes

Student's unpaired t-test with a significance level of $p \leq 0.05$

Supplementary Table S5. Statistical analysis of transformed cycle-threshold (2^{-Ct}) values for comparison of the combination of plate material and cDNA synthesis strategy

Table 5: mean 2^{-Ct} values, standard error of mean, p-values and significance of tissue specific measurements

Tissue	Target	Treatment	2^{-Ct} mean	Standard error of 2^{-Ct} mean	p-value	significant ($p < 0.05$)?
root	*KNL2A*	unspec. x transp.<br>spec. x white	$1.7 \cdot 10^{-06}$<br>$3.3 \cdot 10^{-06}$	$2.8 \cdot 10^{-08}$<br>$1.0 \cdot 10^{-07}$	0.0001	Yes
	KNL2C	unspec. x transp.<br>spec. x white	$7.6 \cdot 10^{-08}$<br>$6.0 \cdot 10^{-07}$	$1.4 \cdot 10^{-09}$<br>$6.9 \cdot 10^{-09}$	<0.0001	Yes
rosette leaf	*KNL2A*	unspec. x transp.<br>spec. x white	$4.4 \cdot 10^{-07}$<br>$9.4 \cdot 10^{-07}$	$1.5 \cdot 10^{-08}$<br>$2.5 \cdot 10^{-08}$	0.0002	Yes
	KNL2C	unspec. x transp.<br>spec. x white	$1.1 \cdot 10^{-08}$<br>$3.5 \cdot 10^{-07}$	$1.1 \cdot 10^{-08}$<br>$2.3 \cdot 10^{-08}$	0.0002	Yes
stem	*KNL2A*	unspec. x transp.<br>spec. x white	$3.5 \cdot 10^{-06}$<br>$6.9 \cdot 10^{-06}$	$7.0 \cdot 10^{-08}$<br>$6.6 \cdot 10^{-08}$	<0.0001	Yes
	KNL2C	unspec. x transp.<br>spec. x white	$4.7 \cdot 10^{-07}$<br>$3.0 \cdot 10^{-06}$	$2.5 \cdot 10^{-08}$<br>$9.5 \cdot 10^{-08}$	<0.0001	Yes
stem leaf	*KNL2A*	unspec. x transp.<br>spec. x white	$5.9 \cdot 10^{-07}$<br>$5.2 \cdot 10^{-07}$	$7.1 \cdot 10^{-08}$<br>$6.4 \cdot 10^{-08}$	0.0541	No
	KNL2C	unspec. x transp.	$7.8 \cdot 10^{-08}$	$7.5 \cdot 10^{-10}$	<0.0001	Yes

		spec. x white	$2.6\cdot10^{-07}$	$4.0\cdot10^{-09}$		
buds	*KNL2A*	unspec. x transp.<br>spec. x white	$7.7\cdot10^{-06}$<br>$1.2\cdot10^{-05}$	$2.0\cdot10^{-07}$<br>$1.4\cdot10^{-06}$	0.0272	Yes
	KNL2C	unspec. x transp.<br>spec. x white	$8.0\cdot10^{-07}$<br>$5.1\cdot10^{-06}$	$2.2\cdot10^{-08}$<br>$3.3\cdot10^{-08}$	<0.0001	Yes
flower	*KNL2A*	unspec. x transp.<br>spec. x white	$5.0\cdot10^{-06}$<br>$8.4\cdot10^{-06}$	$8.8\cdot10^{-08}$<br>$2.3\cdot10^{-07}$	0.0001	Yes
	KNL2C	unspec. x transp.<br>spec. x white	$5.0\cdot10^{-07}$<br>$3.6\cdot10^{-06}$	$2.2\cdot10^{-08}$<br>$6.9\cdot10^{-08}$	<0.0001	Yes
pollen	*KNL2A*	unspec. x transp.<br>spec. x white	$4.9\cdot10^{-08}$<br>$1.9\cdot10^{-08}$	$5.8\cdot10^{-09}$<br>$1.3\cdot10^{-09}$	0.0068	Yes
	KNL2C	unspec. x transp.<br>spec. x white	$4.6\cdot10^{-09}$<br>$1.1\cdot10^{-08}$	$1.7\cdot10^{-09}$<br>$7.0\cdot10^{-10}$	0.0233	Yes
silique	*KNL2A*	unspec. x transp.<br>spec. x white	$7.5\cdot10^{-08}$<br>$2.9\cdot10^{-06}$	$1.3\cdot10^{-08}$<br>$3.8\cdot10^{-08}$	<0.0001	Yes
	KNL2C	unspec. x transp.<br>spec. x white	$1.3\cdot10^{-07}$<br>$1.1\cdot10^{-06}$	$6.0\cdot10^{-09}$<br>$4.1\cdot10^{-08}$	<0.0001	Yes
embryo	*KNL2A*	unspec. x transp.<br>spec. x white	$4.0\cdot10^{-06}$<br>$6.3\cdot10^{-06}$	$1.5\cdot10^{-07}$<br>$2.3\cdot10^{-07}$	0.0011	Yes
	KNL2C	unspec. x transp.<br>spec. x white	$1.1\cdot10^{-09}$<br>$2.9\cdot10^{-06}$	$5.5\cdot10^{-10}$<br>$2.1\cdot10^{-08}$	<0.0001	Yes
seedling	*KNL2A*	unspec. x transp.<br>spec. x white	$2.9\cdot10^{-06}$<br>$5.3\cdot10^{-06}$	$4.0\cdot10^{-08}$<br>$2.5\cdot10^{-07}$	0.0007	Yes
	KNL2C	unspec. x transp.<br>spec. x white	$3.3\cdot10^{-07}$<br>$2.3\cdot10^{-06}$	$3.6\cdot10^{-09}$<br>$1.5\cdot10^{-07}$	0.0002	Yes

Student's unpaired t-test with a significance level of $p \leq 0.05$
